# 

**Published:** 1906-08-15

**Authors:** 


Vol. LX. AUGUST 15, 1906. No. 8.

### THE DENTAL REGISTER

EDITED BY
NELVILLE S. HOFF, D.D.S.,
ANN ARBOR, MICH.

PRICE, ONE DOLLAR A YEAR IN ADVANCE.

Published Monthly by
SAMUEL A. CROCKER & CO.,
Ohio Dental and Surgical Depot.
35 to 39 W. 5th St., Cincinnati, O.

Entered at the Postoffice at Cincinnati, O., as second-class mail matter.

